# Diarrhea and associated factors among under-five children in open defecation free and open defecation rural households of Degem district, Oromia, Ethiopia

**DOI:** 10.3389/fpubh.2024.1480949

**Published:** 2024-12-09

**Authors:** Demelash Dereje, Dejene Hailu, Sisay Abebe Debela, Tamiru Yazew, Fikadu Tolesa, Bogalu Abebe

**Affiliations:** ^1^Department of Public Health, College of Health Sciences, Salale University, Fitche, Ethiopia; ^2^Department of Nursing, College of Health Sciences, Salale University, Fitche, Ethiopia; ^3^Department of Midwifery, College of Health Sciences, Salale University, Fitche, Ethiopia; ^4^Department of Medicine, College of Health Sciences, Salale University, Fitche, Ethiopia

**Keywords:** diarrhea, associated factors, children, open defecation, open defecation free, Ethiopia

## Abstract

**Introduction:**

Diarrheal diseases are the top cause of preventable death, particularly among children under the age of five in developing countries like Ethiopia. Despite the national level of latrine coverage being 61%, diarrhea is responsible for the deaths of half a million children under 5 years annually. Therefore, this study aimed to assess diarrhea and its associated factors among children in open defecation free (ODF) and open defecation (OD) households of Degem district, Oromia, Ethiopia.

**Methods:**

A comparative cross-sectional study was conducted within the community, involving 398 participants (200 from open defecation free [ODF] and 198 from open defecation [ODF] households). The selection of study participants from OD and ODF households was done using a multistage sampling approach. Data input was carried out using Epi Data 3.1, while data analysis would be performed using Statistical Package for Social Sciences (SPSS) version 26 software, employing appropriate testing methods. Statistical significance and the strength of relationships were assessed using odds ratios with a 95% confidence interval.

**Results:**

The prevalence of diarrhea among children in open defecation free and open defecation households was 26 and 38%, respectively. Factors such as children who were not vaccinated for rotavirus, mothers or caregivers did not have diarrhea, mothers or caregivers did not wash their hands at critical times, and individuals with poor latrine utilization were significantly associated with diarrhea among children in ODF households. On the other hand, children who were not vaccinated for rotavirus, not received vitamin A supplementation, mothers or caregivers did not wash their hands at critical times, children lacking access to latrines and children whose their families practice open field solid waste disposal were significantly associated with diarrhea among children in open defecation free households.

**Conclusion:**

The study results indicated that the prevalence of diarrhea among children under five in open defecation areas was notably higher compared to those residing in open defecation free areas. Consequently, it is imperative for all responsible bodies to focus on evidence-based strategies to combat childhood diarrhea and to ensure child health status.

## 1 Introduction

Diarrhea remains a critical global public health issue, resulting in high rates of illness and death among children under 5 years old. According to the World Health Organization (WHO), an estimated 525,000 children in this age group succumb to diarrhea annually, impacting a staggering 1.7 billion individuals worldwide (1). This condition is responsible for one in every nine child fatalities and stands as the second most common cause of death for children under five (2). Notably, Sub-Saharan African countries exhibit a notable prevalence of diarrhea among young children, with this ailment being the primary cause of mortality in this age group, particularly prevalent in South Asia and Sub-Saharan Africa (3). The Ethiopian Demographic and Health Survey (EDHS) report of 2016 indicated that 13% of Ethiopian children experienced diarrhea in the 2 weeks leading up to the survey (4).

In 2017, a report by World Health Organization (WHO)/United Nations Children’s Fund (UNICEF)/Joint Monitoring Programme (JMP) revealed that 670 billion people globally practiced open defecation. Only 40% of urban Africans have access to improved sanitation, and 72% of Ethiopians lack access to these facilities, leading to a high prevalence of diarrhea (5). This has resulted in a significant burden of diarrheal disease in low- and middle-income countries (6). Epidemiologic studies have identified various factors influencing the development of diarrhea in children, with the relative importance of each factor varying based on socioeconomic (7), and environmental factors such as water, sanitation (8), and waste disposal systems (9). Unsafe water, inadequate sanitation, and poor hygiene are responsible for nearly 88% of diarrhea-related deaths. Additionally, approximately 40% of children hospitalized for diarrhea are affected by rotavirus, the most common cause of acute diarrhea (1).

Based on the literature, there is a direct correlation between childhood diarrhea and feeding habits. Early breastfeeding initiation, introduction of complementary feeding, hygiene practices related to complementary meals, and overall hygiene and sanitation during child feeding are all behaviors associated with childhood diarrhea (10). It is worth noting that nutrient-rich breast milk or colostrum can help lower the chances of infectious diseases, specifically acute diarrhea (11).

The prevalence and intensity of diarrhea in Ethiopia are also exacerbated by several factors, such as insufficient access to clean water, inadequate disposal of solid and liquid waste, improper management of human waste, substandard living conditions, and limited access to healthcare services (12).

Numerous studies conducted subsequent to the implementation of Health Service Extension Package (HSEP) revealed an enhancement in the overall health of the community (13). Some of the advancements included the construction and utilization of latrines, promotion of hand hygiene, increased awareness of various health issues, higher vaccine coverage, utilization of maternal services, and promotion of a balanced diet (14). By improving the nation’s water, sanitation, and hygiene, it is estimated that 64,540 children could be saved annually (15). The Community-Led Total Sanitation and Hygiene (CLTSH) approach to preventing diarrheal diseases has also been embraced in Ethiopia. The community-led total sanitation goals encompass eradicating open defecation, using sanitary facilities when necessary, and practicing frequent hand washing (16).

There is limited information on various factors that impact the occurrence of diarrhea, including children’s feeding practices and behavioral factors. Despite efforts in some literature to address the prevalence of diarrhea among open defecation free and open defecation households in relation to socio-demographic, environmental hygiene, and behavioral factors, as well as the use of restrooms and feces disposal for children, there is still debate. It is anticipated that residents in open defecation free households will have lower rates of morbidity and mortality due to diarrhea among under-five individuals compared to residents of open defecation households. Although the health extension program has been in place for decades and rural kebeles are implementing CLTSH, diarrhea remains a serious public health problem for unexplained reasons. Previous studies in different regions of Ethiopia used a cross-sectional design and did not compare the prevalence of diarrhea among children under the age of five and its related variables between open defecation-free and open defecation homes in the research area. Child diarrhea causes might vary by location, district, and environmental factors at the household and community levels. This study result contributes to a better understanding of the fundamentals of open defecation-free status of diarrhea and plays an important role in the proper planning and monitoring of sanitation and hygiene activities and programs that contribute to diarrhea prevention and save the community from its severity. Thus, the this study used a community based comparative cross sectional design to assess and compare the prevalence of diarrhea and its associated variables among children under five in open defecation-free and open defecation households in Degem district, Oromia, Ethiopia.

## 2 Methods

### 2.1 Study area and period

The Degam district is situated between 9°47′29″–9°47′13″N latitude and 38°31′09″–38°32′50″E longitudes, approximately 131 km North of Addis Ababa. Hambiso serves as the capital town for Degem district, housing the district’s administrative office. According to the 2007 national census, the total population of this district was 99,143, with 49,205 men and 49,938 women; 6.12% of the population were urban dwellers. The number of under-5 children in the district was 23,848. Additionally, there are 6 health centers, 20 health posts, 1 medium private clinic, 2 lower private clinics, and 1 private drug store in Degem district. The study was employed from August 21 to October 21, 2023.

### 2.2 Study design and population

Community based comparative cross-sectional study design was conducted. The source population for this study consisted of all mothers/caregivers with children under five residing in open defecation free and open defecation kebeles of Degem district, North Shoa Zone, Oromia region, Ethiopia. The study involved children who were living in the study area with their mothers or caregivers for over 6 months in the ODF and OD kebeles. Children whose mothers or caregivers were seriously ill, as well as children taking medication, were not included in the study.

### 2.3 Sample size determination and sampling procedures

The sample size was calculated using the STATCALC tool within EPI INFO statistical software version 7. The calculation took into account a prevalence of 19.3% for diarrheal disease among households in open defecation free households (16) and 36.2% in open defecation households (17). Other factors considered were a 95% confidence interval, 80% power, a margin of error of 5%, a 1:1 ratio of ODF to OD households, and a design effect of 1.5. Therefore, by considering design effect of 1.5 and 10% non-response rate, the final total sample size was 400 households, (200 samples from open defecation free and 200 from open defecation households).

The study area was deliberately chosen. A multistage sampling technique was utilized. Out of the 18 rural Kebeles in the Degem district, six Kebeles are ODF while 12 Kebeles are OD. From these 18 Kebeles, three ODF Kebeles and six OD Kebeles were selected through a lottery method using the list of each Kebele as a sampling frame, and households with children under five were distributed proportionally. This resulted in 200 households from the three ODF Kebeles and 200 households from the six OD Kebeles, making a total sample size of 400. A systematic sampling method was then employed to select households with under-5 children from each Kebele. To determine the interval (*k*), the number of under-5 children was divided by the proportional sample size in each Kebele. Therefore, *k* = 2,975/200 = 15 for ODF households and *k* = 8,161/200 = 41 for OD households. The first household was selected using a lottery method, with the list of households with under-5 children obtained from family folders collected by health extension workers.

### 2.4 Data collection and management

A structured interviewer-administered questionnaire was modified from various studies conducted in the country regarding the subject, along with an observational checklist employed to assess latrine usage (12, 18–23). The questionnaire was meticulously translated from English to Afaan Oromoo and subsequently back to English by two language specialists proficient in both languages. Six data collectors, all of whom had completed high school or attained a higher level of education, were trained for this task. Additionally, two health professionals with degrees were appointed to oversee the data collection process. Data was gathered from selected study participants through home visits. Complex terminologies were clarified to respondents in the local language to minimize discrepancies in the information provided. Prior to actual data collection, a pre-test was carried out on 5% of the study participants with similar socio-demographic characteristics in Debre Libanos district to validate the questionnaire items. Any necessary revisions to the questionnaire were made based on the pre-test results. Following data collection, a double data entry process was conducted on the computer simultaneously to ensure data consistency.

### 2.5 Study variables

The dependent variable included status of under-five diarrheas. Independent variables were socio-economic and demographic, environmental, maternal and child health and behavioral factors.

### 2.6 Operational definitions

**Infant:** Child under the age of 1 year (5).

**Under-five children**: Refers to all children from 0 to 59 months (5).

**Diarrhea:** the proportion of children under the age of five who had three or more loose or watery stools every 24 h during the previous 2 weeks of data collection, as reported by the children’s mothers/caregivers (24).

### 2.7 Statistical analysis

The data gathered from the questionnaire was coded and inputted into Epi Data version 3.1, then cleaned to ensure accurate and consistent entry of all variables. The cleaned data was then transferred to SPSS windows version 26 for analysis. The results were presented using tables and figures, and descriptive analysis was conducted by calculating percentages, frequencies, and means. Additionally, frequency distribution of dependent and independent variables was examined. The presence of multi-collinearity was assessed using the Variance Inflation Factor (VIF), which was found to be between 1 and 5 for all independent variables. Bivariate logistic regression analysis was carried out, and explanatory variables with a *p* value ≤0.2 were included in the final regression model. Multivariable logistic regression analysis was then performed to determine the associations of selected variables with the dependent variables, and predictors were identified through adjusted odds ratio (AOR) with a 95% confidence interval and *p* < 0.0.05. Model fitness was evaluated using the Hosmer Lemeshow test, and a *p* value >0.05 was obtained.

## 3 Results

### 3.1 Socio-demographic and economic characteristics

A total of 398 individuals participated in the interviews, comprising 200 from the NOD group and 198 from the OD group, resulting in a response rate of 398 (99.5%). Among the respondents, 170 females (85%) were from the NOD group, while 161 females (81.3%) were from the OD households (Table 1). In terms of age distribution, 122 respondents (61%) from ODF and 115 respondents (58%) from OD households fell within the 25–34 years age range. The average age was calculated to be 30.78 ± 5.98 SD years for those in ODF households and 30.55 ± 5.704 years for those in OD kebeles. Additionally, 174 respondents (87%) in ODF and 160 respondents (81%) in OD households identified as the biological mothers of the index child. The predominant religious affiliation among respondents was Orthodox Christianity, with 190 individuals (95%) in ODF and 169 individuals (85%) in OD households identifying as such. Furthermore, regardless of their educational qualifications, 103 respondents (51.5%) from ODF and 81 respondents (40.9%) from OD households were illiterate. The majority of respondents were married, with 195 individuals (97.5%) in ODF and 191 individuals (96.4%) in OD households indicating their marital status (Table 1).

**Table 1:** Socio-demographic characteristics of the study participants of Degem district, Oromia, Ethiopia in 2023.

Variables	Category	ODF (*N* = 200)	OD (*N* = 198)	Chi-square	*p* value
		*n* (%)	*n* (%)		
Sex of respondent	Male	30 (15)	37 (18.7)	0.173	0.677
	Female	170 (85)	161 (81.3)		
Age of respondent	15–24	21 (10.5)	28 (14.1)	3.854	0.146
	25–34	122 (61)	115 (58.1)		
	≥35	57 (28.5)	55 (27.8)		
Relationship of respondent and child	Mother	174 (87)	160 (80.8)	0.105	0.746
	Father	25 (12.5)	38 (19.2)		
Religion f respondent	Orthodox	190 (95)	169 (85.4)	1.38	0.848
	Protestant	9 (4.5)	24 (12.1)		
	Muslim	1 (0.5)	3 (1.5)		
Educational status of respondent	Unable to read and write	103 (51.5)	81 (40.9)		
	Informal education	1 (0.5)	2 (1)	1.654	0.799
	Grade 1–8	76 (38)	92 (46.5)		
	Grade 9–12	18 (9)	20 (10.1)		
	Grade 12 and above	2 (1)	3 (1.50)		
Marital status of respondent	Married	195 (97.5)	191 (96.4)		
	Single	2 (1)	3 (1.5)	0.87	0.62
	Widowed	3 (1.5)	2 (1)		
	Divorced (legal)	0 (0)	2 (1)		
Monthly income	<1,000	18 (9)	11 (5.6)	5.589	0.133
	1,000–1,999	48 (24)	43 (21.7)		
	2,000–2,999	67 (33.5)	65 (32.8)		
	≥3,000	67 (33.5)	79 (39.9)		
Total family members	3–6	185 (92.5)	186 (93.6)	6. 326	0.276
	>6	15 (7.5)	12 (6.1)		
Total under five children	1–2	199 (99.5)	195 (98.5)	0.769	0.681
	≥3	1 (0.5)	3 (1.5)		

ODF, Open defecation free; OD, Open defecation Socio-demographic characteristics of under five children.

The majority of children included in the study were male, with 111 (55.5%) in ODF and 126 (63.6%) in OD households falling into this category (Table 2). In the age group of 24 to 59 months, there were 140 (70%) children in ODF and 141 (71.2%) in OD households. The mean age of children was 29 ± 14.088 SD months in ODF and 30.55 ± 5.707 SD months in OD households. Those children who lived with their biological mother were 194 (97%) children in ODF and 195 (98.5%) children in OD. The study also revealed that 77 (38.5%) children in ODF and 85 (42.9%) children in OD households were born first (Table 2).

**Table 2:** Socio-demographic characteristics of under-five children of Degem district, Oromia, Ethiopia in 2023.

Variables	Category	ODF (*n* = 200)	OD (*n* = 198)	Chi square	*p* value
		*n* (%)	*n* (%)		
Sex of index child	Male	111 (55.5)	126 (63.6)	0.094	0.759
	Female	89 (44.5)	72 (36.4)		
Age of index child	0–5	7 (3.5)	4 (2)	3.188	0.364
	6–11	17 (8.5)	21 (10.6)		
	12–23	36 (18)	32 (16.2)	4.688	0.096
	24–59	140 (70)	141 (71.2)		
Birth order of child	1st	77 (38.5)	85 (42.9)		
	2nd	61 (30.5)	57 (28.8)	9.221	0.101
	≥3rd	62 (31)	56 (28.3)		
Child lives with whom	Mother	194 (97)	195 (98.5)	0.723	0.395
	Caregiver	6 (3)	3 (1.5)		

ODF, Open defecation free; OD, Open defecation.

### 3.2 Feeding practice of children

According to the latest survey, the majority of children in ODF and OD households breastfed, with 88 and 93.9%, respectively. Among those who breastfed, 70.5% in ODF and 82.8% in OD households started within 1 h of birth, and most had no history of pre-lactation feeding, with 90.0% in ODF and 92.9% in OD. Additionally, 50.7% in ODF and 30.8% in OD households exclusively breastfed their index child during the first 6 months of life (Table 3).

**Table 3:** Feeding practices of children of under five children of Degem district, Oromia, Ethiopia in 2023.

Variables	Category	ODF		OD		Chi square	*p* value
		Frequency (*n*)	Percent (%)	Frequency (*n*)	Percent (%)		
Breast feeding	Yes	176	88	186	93.9	0.266	0.606
	No	24	12	12	6.1		
Breast feeding initiation time within 1 h	Yes	141	70.5	164	82.8	1.568	0.211
	No	59	29.5	34	17.2		
Pre-lacteal feeding	Yes	20	10.0	14	7.1	0.260	0.610
	No	180	90.0	184	92.9		
Exclusive breast feeding within 1st 6 months	Yes	51	26.3	61	30.8	4.119	0.128
	No	143	73.7	132	67.2		
Complementary feeding at 6th months	Yes	16	8.2	51	25.8	4.608	0.100
	No	178	91.8	142	71.7		
Have the child feed the breast minimum for 2 year	Yes	117	70.5	98	49.5	1.414	0.493
	No	49	29.5	48	24.2		
Have the child feed by bottle	Yes	81	40.5	77	38.9	11.827	0.001
	No	119	59.5	121	61.1		
Have the child feed uncooked foods	Yes	40	20.6	41	20.7	0.023	0.989
	No	154	79.4	151	76.3		
Was the child feed cooked foods immediately after cooking	Yes	139	72	137	69.2	2.132	0.344
	No	54	28	57	28.8		
Was the child feed unwashed fruits	Yes	106	54	90	45.5	0.073	0.964
	No	88	45.4	103	52.0		
Was feeding Utensils washed with soap and water	Yes	138	69.0	87	43.9	2.381	0.304
	No	62	31.0	104	52.5		
Was the child drink treated water	Yes	64	33.5	23	11.6	9.358	0.009
	No	127	66.5	170	85.9		

ODF, Open defecation free; OD, Open defecation.

**Figure 1:**
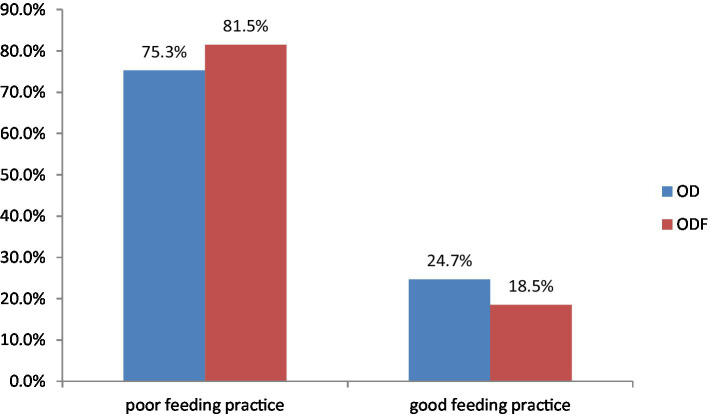
Feeding practice of children under 5 years in Degem district, Oromia, Ethiopia in 2023. ODF, Open defecation free; OD, Open defecation.

Overall, out of the 11 questions regarding feeding practices, only 18.5% of participants in ODF and 24.7% in OD households demonstrated good feeding practices (Figure 1).

### 3.3 Child and mother/caregiver health related characteristics

**Figure 2:**
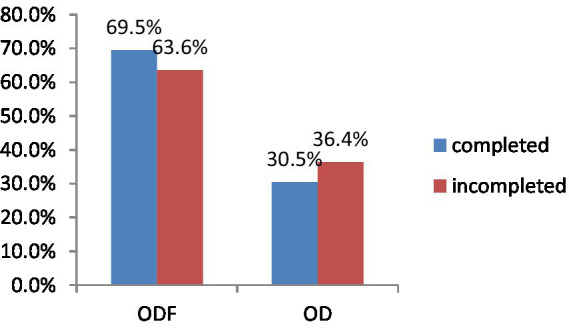
Immunization statuses of under-five children of Degem district, Oromia, Ethiopia in 2023.

The study finding revealed variations in vaccination records among children five in ODF and OD households. Specifically, 139 (69.5%) children from ODF and 126 (63.6%) from OD were included in this study (Figure 2).

**Figure 3:**
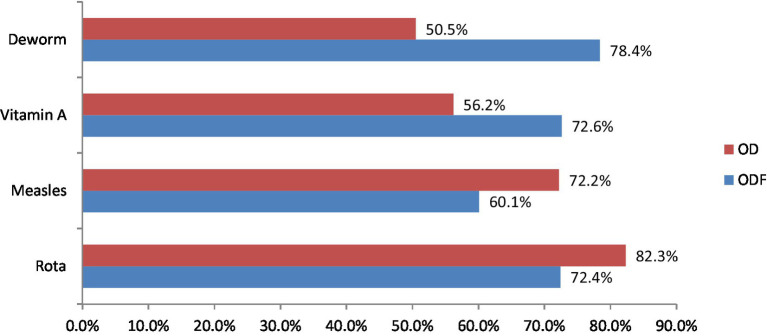
Child health related characteristics in Degem district, Oromia, Ethiopia in 2023. ODF, Open defecation free; OD, Open defecation.

The study finding also indicates that in the ODF and OD households, the history of vitamin A supplementation among children was recorded at 138 (72.6%) and 109 (56.2%), respectively. In contrast, the immunization rates against measles were 110 (60.1%) for ODF and 143 (72.2%) for OD households (Figure 3). Furthermore, the study revealed that 144 (72.4%) of children in ODF and 163 (82.3%) in OD had received vaccinations for the Rota virus. However, only 127 (78.4%) of children in ODF and 100 (50.5%) in OD had been administered deworming medications (Figure 3).

### 3.4 Environmental characteristics of respondent household

The roofing material used in the majority of households in ODF and OD households was iron, with 81 and 84.3% of respondents reporting this, respectively. However, most of the floors in their houses were made of mud. In ODF, 43.7 and 45.7% of households had iron roofs. Nearly all households in ODF (99.9%) and around 90.4% in OD households lived in homes with three rooms. The survey also revealed that 71.5% of ODF households and 60.1% of OD households did not share their living space with cattle. Only 45.5% of ODF and 13.3% of OD households had access to drinking water from a protected source. However, the majority of households in ODF (84%) and OD households (86.4%) could obtain water within 30 min. A smaller percentage of ODF households (30.5%) and OD households (56.6%) disposed of solid waste in open fields, while more households in ODF (55.5%) and OD households (74.7%) disposed of liquid waste in open fields. Only 12.7% of ODF households with latrines had hand washing facilities nearby, compared to just 2.1% of OD households with latrines (Table 4).

**Table 4:** Environmental characteristics of respondents of Degem district, Oromia, Ethiopia in 2023.

Variables	Category	ODF kebeles		OD kebeles		Chi square	*p*-value
		Frequency (*n*)	Percent (%)	Frequency (*n*)	Percent (%)		
Roof	Thatched	38	19.0	31	15.7	0.655	0.418
	Iron	162	81.0	167	84.3		
Floor	Mud	174	87.0	182	91.9	8.315	0.04
	Cement	26	13.0	16	8.1		
Number of rooms	<3	199	99.5	179	90.4	0.456	0.499
	≥3	1	0.5	19	9.6		
Livestock	Yes	57	28.5	79	39.9	2.214	0.137
	No	143	71.5	119	60.1		
Source of drinking water	Protected	91	45.5	26	13.1	3.051	0.081
	Not protected	109	54.5	172	86.9		
Time taken to reach source of water	≤30 min	168	84	171	86.4	2.652	0.103
	>30 min	9	4.5	172	86.9		
Place of solid waste disposal	Open field	44	22%	73	36.9	9.044	0.011
	Pit/Burning	156	78%	112	56.6		
Place of liquid waste disposal	Open field	111	55.5	148	74.7	0.002	0.968
	Pit	89	44.5	50	25.3		
Have you latrine	Yes	177	88.5	115	58.1	3.464	0.063
	No	23	11.5	83	41.9		
Hand washing facility near the toilet	Yes	25	14.1	6	4.8	0.002	0.968
	No	152	85.9	99	95.2		
Have you hand wash after using toilet	Yes	108	54.0	73	36.9	12.937	0.000
	No	92	46.0	125	63.1		
Hand wash after cleaning child	Yes	156	78.0	100	50.5	11.285	0.001
	No	44	22.0	98	49.5		
Hand wash after any cleaning activity	Yes	189	94.5	167	84.3	12.583	0.000
	No	11	5.5	31	15.7		
Hand wash before preparing food	Yes	192	96.0	193	97.5	5.194	0.023
	No	8	4.0	5	2.5		
Hand wash before meal	Yes	199	99.5	194	98.0	3.409	0.065
	No	1	0.5	4	2.0		
Hand wash before breast feeding	Yes	19	9.5	16	8.1	7.657	0.022
	No	181	90.5	182	91.9		
Hand washing method	Water and soap	125	62.5	74	37.4	14.339	0.001
	Water and ash	22	11.0	41	20.7		
	Water only	53	26.5	83	41.9		

ODF, Open defecation free; OD, Open defecation.

### 3.5 Hand washing practice at critical time

**Figure 4:**
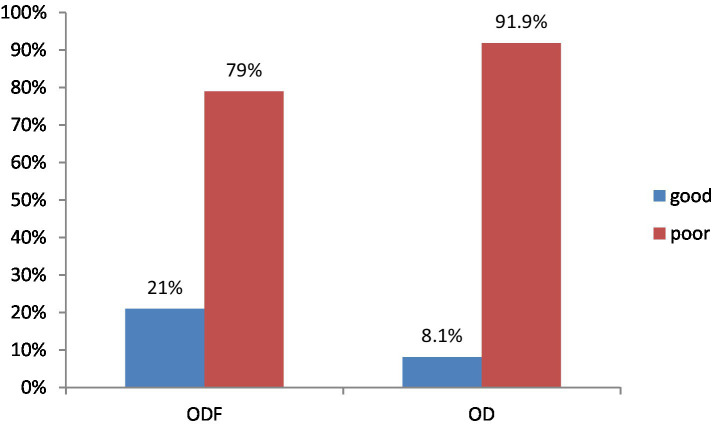
Hand washing practice at critical time of Degem district, Oromia, Ethiopia in 2023. ODF, Open defecation free; OD, Open defecation.

Hand washing was substantially better in ODF (21%) households than those in OD (8.1%) (Figure 4).

### 3.6 Latrine coverage and utilization

**Figure 5:**
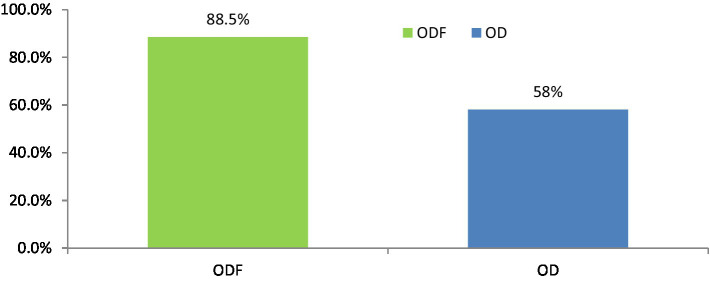
Latrine coverage status of study participants in Degem district, Oromia, Ethiopia in 2023. ODF, Open defecation free; OD, Open defecation.

**Figure 6:**
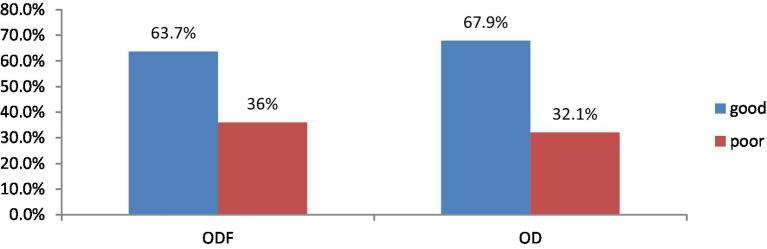
Latrine utilization of study participants of Degem district, Oromia, Ethiopia in 2023. ODF, Open defecation free; OD, Open defecation.

This survey found that 96.5% of ODF and 54% of OD households have latrines. From those have latrine; good latrine utilization was more practiced by ODF households, 63.7% than those households in OD 67.9% (Figures 5, 6).

### 3.7 Prevalence of diarrhea

**Figure 7:**
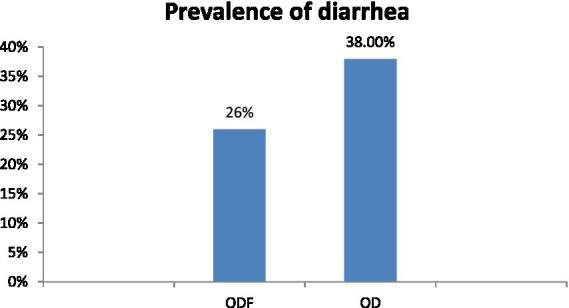
Prevalence of diarrhea among children in open defecation free and open defecation rural households of Degam district, Oromia, Ethiopia.

The diarrheal disease in children under-five was more common among open defecation households compared to those households in open defecation free. The occurrence of previous two-week’s diarrheal morbidity among children under the age of five in ODF households was 26% which was lower than the prevalence among children in OD households, 38% (Figure 7).

### 3.8 Factors associated with under five children’s diarrhea in open defecation free and open defecation rural households

In this study, the rotavirus vaccine, presence of diarrhea in mothers, hand washing at critical times, and latrine utilization were included in the model as predictors with a significant association with the occurrence of diarrhea in children under five. Children who were not vaccinated for rotavirus were 3.33 times more likely to develop diarrhea than those who were vaccinated (AOR = 3.33, 95% CI [1.14–9.7]). Additionally, under-five children whose mothers or caregivers did not have diarrhea were 75% less likely to acquire diarrhea than those who did (AOR = 0.25, 95% CI = [0.065–0.95]). Furthermore, children whose mothers or caregivers did not wash their hands at critical times were 5.3 times more likely to experience diarrhea than those who did wash their hands at critical times (AOR = 5.3, 95% CI = [1.77–15.56]). The research also revealed that individuals with poor latrine utilization were 3.3 times more likely to develop diarrhea than those with good latrine utilization (AOR = 3.3, 65% CI [1.09–10]) (Table 5).

**Table 5:** Factors associated with diarrhea in open defecation free of Degem district, Oromia, Ethiopia in 2023.

Variables	Category	ODF households				
		Presence of diarrhea		COR (95%CI)	AOR (95%CI)	*p*-value
		Yes (Frequency, %)	No (Frequency, %)			
Rota vaccine	No*	15 (57.7)	40 (23.1)	4.53 (1.93–10.67)	3.33 (1.14–9.7)	0.028
	Yes^1^	11 (42.3)	133 (76.9)			
Presence of diarrhea in mother	No*	18 (69.2)	156 (90.8)	0.23 (0.08–0.61)	0.25 (0.065–0.95)	0.042
	Yes^1^	8 (30.8)	16 (9.2)			
Hand wash at critical time	Poor*	14 (53.8)	28 (16.1)	6.08 (2.55–14.53)	5.3 (1.77–15.56)	0.003
	Good^1^	12 (46.2)	146 (83.9)			
Child stool disposing site	Open field	16 (61.5)	53 (30.5)	3.65 (1.56–8.58)	2.56 (0.89–7.422)	0.082
	Latrine^1^	10 (36.5)	121 (69.5)			
Latrine utilization	Poor*	17 (70.8)	53 (31.4)	5.31 (2.08–13.6)	3.3 (1.09–10)	0.034
	Good^1^	7 (29.2)	116 (68.6)			
OD households						
Rota	No*	18 (47.4)	17 (10.6)	7.57 (3.36–17.04)	5.97 (2.34–15.2)	0.0.001
	Yes^1^	20 (52.6)	143 (89.4)			
Vitamin A	No*	28 (73.7)	57 (36.5)	4.86 (2.2–10.74)	4.81 (1.58–12.36)	0.001*
	Yes^1^	10 (26.3)	99 (63.5)			
Place of solid waste dispose	Open field*	31 (81.6)	112 (70)	4.32 (1.8–10.4)	4.82 (2.65–14.12)	0.004*
	Pit/burn^1^	7 (18.4)	48 (30)			
Availability of latrine	No*	26 (68.4)	57 (35.7)	3.91 (1.84–8.34)	2.66 (1.09–6.45)	0.03*
	Yes^1^	12 (31.6)	103 (64.3)			
Hand washing at critical time	Poor	32 (84.2)	150 (93.8)	2.81 (0.95–8.3)	6.78 (1.65–27.93)	0.008*
	Good	6 (15.8)	10 (6.2)			
Pooled analysis						
Bottle feed	No	28 (43.7)	212 (63.5)	0.448 (0.26–0.77)	0.42 (0.17–1.04)	0.063
	Yes^1^	36 (56.3)	122 (36.5)			
Rota vaccine	No*	33 (51.6)	57 (57.1)	5.15 (2.92–9.09)	5.6 (2.19–14.29)	0.0001
	Yes^1^	31 (48.4)	276 (42.9)			
Vitamin A	No	40 (62.5)	223 (69.7)	3.83 (2.19–6.71)	2.44 (0.98–6.09)	0.056
	Yes^1^	24 (37.5)	97 (30.3)			
Deworm	No	38 (62.3)	95 (31.8)	3.54 (2–6.29)	2.51 (0.996.6.35)	0.051
	Yes^1^	23 (37.7)	204 (68.2)			
Diarrhea in mother/caregiver	No*	35 (54.7)	288 (86.2)	0.19 (0.108–0.34)	0.24 (0.081–0.67)	0.004
	Yes^1^	29 (45.3)	46 (13.8)			
Solid waste disposing site	Open field*	42 (65.6)	114 (34.1)	3.64 (2.1–6.47)	2.95 (1.18–7.38)	0.021
	Pit/burn^1^	22 (34.4)	220 (65.9)			
Latrine availability	No*	32 (50)	74 (22.2)	3.51 (2.02–6.11)	10.78 (2.91–39.94)	0.000
	Yes^1^	32 (50)	260 (77.8)			

*p-value < 0.05; ^1^reference; ODF, Open defecation free; OD, Open defecation.

Following the adjustment for confounding variables through multivariable logistic regression analysis, the factors of rotavirus vaccination, vitamin A supplementation, solid waste disposal methods, hand washing at critical times, and availability of latrines were identified as significant predictors associated with the incidence of diarrhea in children under 5 years of age. Specifically, unvaccinated children were found to be 5.97 times more likely to experience diarrhea compared to their vaccinated counterparts [AOR = 5.97, 95% CI = (2.34–15.2)]. Additionally, children who did not receive vitamin A supplementation were 4.81 times more likely to suffer from diarrhea than those who did [AOR = 4.81, 95% CI = (1.58–12.36)]. Furthermore, participants whose families disposed of solid waste in open fields were 4.82 times more likely to contract diarrhea compared to those who utilized latrines for waste disposal [AOR = 4.82, 95% CI = (2.65–14.12)]. Children lacking access to latrines were 2.66 times more likely to develop diarrhea than those with latrine access [AOR = 2.66, 95% CI = (1.09–6.45)]. Lastly, children exhibiting poor hand washing practices at critical times were 6.78 times more likely to acquire diarrhea compared to those with good hand washing practices [AOR = 6.78, 95% CI = (1.65–27.93)] (Table 5).

## 4 Discussion

The occurrence of diarrhea in children under the age of five was 13% in ODF and 19.2% in OD. Factors such as Rota vaccination, diarrhea in mother/caregiver, hand washing at critical times, and latrine utilization in ODF, as well as Rota vaccine, Vitamin A supplementation, place of solid waste disposal, latrine availability, and hand washing at critical times in OD households were found to be independently linked to diarrheal disease in children under the age of 5. This research revealed that children residing in OD households had a slightly higher prevalence of diarrhea compared to those in ODF households. This difference could be attributed to variations in CLTS implementation in ODF sub-districts, as well as variances in household knowledge of environmental sanitation across sub-districts. Additionally, variations in sanitation facility coverage between these two sub-districts could be the reason behind the difference in diarrhea prevalence. Previous studies in North Central Ethiopia (17), and a multicenter study in Kenya (25) both identified open defecation as a risk factor for moderate to severe diarrheal illnesses.

The overall prevalence of diarrhea in the study area was found to be 16.1%, which exceeds the combined prevalence rates reported in various studies, including Tanzania (12.33%) (26), as well as Bangladesh (5.71%) (27). However, this figure is lower than the prevalence reported in Southern Ethiopia (33.7%) (28), and a joint study conducted in Cameroon (23.8%) (29). Furthermore, it is also less than the prevalence observed in Jimma zone (23.1%) (30). The observed differences may be attributed to variations in socio-demographic factors, geographical conditions, climate, practices related to feces disposal, access to water, hand hygiene practices, and dietary habits.

The findings revealed that the prevalence of diarrhea among children under five was lower in ODF households in Degem district compared to a study done in North central Ethiopia, 19.3% (16) and Southern Ethiopia, with rates of 18.9% (31). The lower rate observed in our study could be attributed to effective monitoring, follow-up, and the implementation of open defecation-free households after the CLTSH intervention. Another study conducted in Yaya Gulele District in Ethiopia reported a 13.4% occurrence of diarrheal disease among children under five, which is consistent with our survey (32). However, this result is higher than a previous study conducted in Ethiopia (9.9%) (33), Kenya (11.1%) (21), and India (2.72%) (34). This disparity may be explained by the fact that ODF households in our study have not yet achieved 100% latrine coverage and still practice open defecation.

The likelihood of diarrhea in children under the age of five was higher among those whose mothers had diarrhea in the 2 weeks prior to the survey, compared to those whose mothers did not. A similar finding was reported in a previous study conducted in South Ethiopia and (12) and north central Ethiopia (16).

Children whose mothers or caregivers practiced proper hand washing during a critical period were less prone to developing diarrhea than those whose hand washing practices were poor. This aligns with a previous study conducted in the Region of Oromia, Ethiopia (35). Households with inadequate latrine utilization were at a higher risk of diarrhea compared to households with good latrine utilization. This finding is in line with research conducted in Northern Ethiopia (16), Southern Ethiopia (25), Jima zone (23), and Southwest Ethiopia (36).

The study findings revealed that the prevalence of two-week under five diarrhea morbidity among children under five living in OD households was 19.2%, which is lower than the rates found in other areas such as Yaya Gulele, Ethiopia, 36.3% (32), Kersa, Ethiopia, 22.2% (23), Southern Ethiopia, 36.2% (24), North central Ethiopia, 40.5% (16), Kenya, 21.6% (37), and Mali, 24% (34). These differences in prevalence may be attributed to variations in the performance and implementation of CLTSH packages across different countries. Another study conducted in northern Ethiopia, found that the prevalence of diarrheal sickness in children under the age of five was 20.2%, which is nearly equivalent to our study location (12).

The study also found that vitamin A supplementation for children under the age of five was an independent predictor of childhood diarrhea. In this study, the odds of diarrhea among under-five children from non-model families who had not received vitamin A supplements within 6 months of the survey were approximately three times higher than the odds of childhood diarrhea among under-five children from non-model families who had received vitamin A supplements during the same time period. This finding was consistent with other similar studies conducted in, West Ethiopia (10), South Ethiopia (13), and the Global Burden of Disease Study 2016 (38).

Rotavirus is recognized as a significant contributor to diarrhea in children under five, which can be prevented through immunization against the virus. The presence of a Rotavirus vaccine emerged as a crucial factor in predicting instances of childhood diarrhea in this study. Children who received the Rotavirus vaccine exhibited a lower likelihood of experiencing diarrhea in the OD households compared to their unvaccinated counterparts. This finding aligns with earlier research examining the correlation between diarrhea in children under five and their vaccination status against Rotavirus, including studies conducted in West Ethiopia (10), northern Ethiopia (12), and South Ethiopia (13).

Furthermore, diarrhea among children under five was found to be more prevalent in families that employed inadequate solid waste disposal methods, in contrast to those that practiced proper waste management. This observation is consistent with findings reported in rural areas of Dangla District in northern Ethiopia in 2017 (12), Northwest Ethiopia (20), and Southwest Ethiopia (36).

Additionally, the results of this study revealed that households equipped with no latrines were more susceptible to diarrhea than those household who had latrine utilization. This observation corroborates previous research conducted in the rural community of Sheko District in Southwest Ethiopia (36). This may be attributed to the fact that effective management of diarrhea can diminish its morbidity.

## 5 Conclusion

The prevalence of diarrhea among children under 5 years is slightly higher in open defection households compared to open defecation free households in Degem district, but the association is not significant. The key factors influencing the occurrence of under-five diarrhea in open defecation free households included Rota vaccination, maternal/caregiver diarrhea, hand washing at critical times, and latrine use. On the other hand, the primary predictors affecting the prevalence of under-five diarrhea in open defecation households were Rota virus vaccination, vitamin A supplementation, solid waste disposal location, latrine availability, and hand washing at critical times. Therefore, there is a need to strengthen aggressive health education and promotion for mother/caregivers on hygiene and environmental sanitation activities to tackle predictors of child diarrhea at household and community levels as well as to alleviate the burden of diarrhea morbidity in the study area.

## 6 Limitation and strengths of the study

There is a lack of research on diarrhea among children under 5 years old in ODF and OD households within rural communities, leading to an inadequate discussion section. Additionally, the cross-sectional study design used in this investigation hindered the ability to establish a temporal relationship between diarrhea prevalence and its associated factors. The study’s strength lies in being potentially the first of its kind in the area, aiming to explore variables linked to diarrhea in both ODF and OD households. The assessment of latrine usage through observation and addressing hand washing practices during critical times are notable aspects of the study.

## Data Availability

The original contributions presented in the study are included in the article/supplementary material, further inquiries can be directed to the corresponding author.
